# Artificial intelligence-powered prediction of diabetic complications: from clinical data to molecular omics

**DOI:** 10.1093/bib/bbag083

**Published:** 2026-02-26

**Authors:** Xueqin Xie, Changchun Wu, Ziru Huang, Yuwei Zhou, Jian Huang, Fuying Dao, Dan Yan, Kejun Deng, Hao Lyu, Caiyi Ma, Hao Lin

**Affiliations:** Department of Clinical Laboratory, Sichuan Clinical Research Center for Cancer, Sichuan Cancer Hospital & Institute, Sichuan Cancer Center, School of Life Science and Technology, University of Electronic Science and Technology of China, 2006 Xiyuan Avenue, West Hi-Tech Zone, Chengdu 610054, China; Department of Clinical Laboratory, Sichuan Clinical Research Center for Cancer, Sichuan Cancer Hospital & Institute, Sichuan Cancer Center, School of Life Science and Technology, University of Electronic Science and Technology of China, 2006 Xiyuan Avenue, West Hi-Tech Zone, Chengdu 610054, China; Department of Clinical Laboratory, Sichuan Clinical Research Center for Cancer, Sichuan Cancer Hospital & Institute, Sichuan Cancer Center, School of Life Science and Technology, University of Electronic Science and Technology of China, 2006 Xiyuan Avenue, West Hi-Tech Zone, Chengdu 610054, China; Department of Clinical Laboratory, Sichuan Clinical Research Center for Cancer, Sichuan Cancer Hospital & Institute, Sichuan Cancer Center, School of Life Science and Technology, University of Electronic Science and Technology of China, 2006 Xiyuan Avenue, West Hi-Tech Zone, Chengdu 610054, China; Department of Clinical Laboratory, Sichuan Clinical Research Center for Cancer, Sichuan Cancer Hospital & Institute, Sichuan Cancer Center, School of Life Science and Technology, University of Electronic Science and Technology of China, 2006 Xiyuan Avenue, West Hi-Tech Zone, Chengdu 610054, China; School of Biological Sciences, Nanyang Technological University, 50 Nanyang Avenue, Singapore 639798, Singapore; Beijing Friendship Hospital, Capital Medical University, 10 Xitoutiao Road, Youanmenwai Avenue, Fengtai District, Beijing 100069, China; Department of Clinical Laboratory, Sichuan Clinical Research Center for Cancer, Sichuan Cancer Hospital & Institute, Sichuan Cancer Center, School of Life Science and Technology, University of Electronic Science and Technology of China, 2006 Xiyuan Avenue, West Hi-Tech Zone, Chengdu 610054, China; Department of Clinical Laboratory, Sichuan Clinical Research Center for Cancer, Sichuan Cancer Hospital & Institute, Sichuan Cancer Center, School of Life Science and Technology, University of Electronic Science and Technology of China, 2006 Xiyuan Avenue, West Hi-Tech Zone, Chengdu 610054, China; School of Computer Science and Technology, Aba Teachers College, Shuimo Town, Wenchuan 623002, China; Department of Clinical Laboratory, Sichuan Clinical Research Center for Cancer, Sichuan Cancer Hospital & Institute, Sichuan Cancer Center, School of Life Science and Technology, University of Electronic Science and Technology of China, 2006 Xiyuan Avenue, West Hi-Tech Zone, Chengdu 610054, China

**Keywords:** diabetic complications, artificial intelligence, risk prediction, large language models, machine learning

## Abstract

Diabetic complications are a major cause of disability and mortality among patients, and early identification of high-risk individuals is essential for precision prevention and management. In recent years, the rapid advancement of artificial intelligence (AI) has provided transformative tools for risk prediction and clinical decision support in diabetes care. In this narrative review, we systematically surveyed studies published between January 2015 and June 2025 in PubMed, Web of Science, and Scopus that applied AI-based predictive modeling for three major diabetic complications: diabetic retinopathy (DR), diabetic nephropathy (DN), and diabetic cardiovascular disease (CVD). A total of 58 studies were included, encompassing models based on clinical features, molecular omics, medical imaging, and multimodal data integration. Cross-scale and multimodal data fusion has emerged as a promising new paradigm, demonstrating improved predictive performance over single-modality approaches in three major diabetic complications. We also summarize the evolution from traditional machine learning to deep learning and, more recently, to large language models and agent-based systems, comparing their methodological characteristics, strengths, and suitable application scenarios. Finally, we proposed an actionable six-step framework and clinical translation pathway for AI in diabetic complications, outlining key steps from data curation and model development to validation, regulatory compliance, and real-world implementation. Together, these insights provide a roadmap toward developing robust, transparent, and clinically deployable AI systems capable of transforming the prevention and management of diabetic complications.

## Introduction

Diabetes mellitus (DM), a rapidly growing global chronic disease, has positioned early prediction and precision management of its complications a central focus in medical research. According to the International Diabetes Federation, ~537 million adults worldwide live with diabetes, with most progressing to at least one severe complication during their disease course [1, 2]. These complications significantly impair quality of life, increase healthcare costs, and impose a heavy socioeconomic burden [3]. Among diabetes-related complications, diabetic retinopathy (DR), diabetic nephropathy (DN), and diabetic cardiovascular disease (CVD) form a ‘triple threat’ in diabetic care due to their high prevalence, devastating consequences, and complex pathological mechanisms. DR remains the leading cause of blindness in working adults and the elderly [4, 5]. DN is a major contributor to end-stage kidney disease (ESKD) and is closely associated with increased mortality [6, 7], while CVD accounts for over 50% of diabetes-related deaths [8, 9]. Although these complications arise from distinct pathological pathways, early prediction is essential for improving outcomes, prompting the development of predictive models based on different feature modalities [10–12].

Recent breakthroughs in artificial intelligence (AI) have significantly advanced the development of precision prediction models for diabetic complications [13, 14]. Current models primarily use clinical features due to data availability. In DR, machine learning (ML) models integrating retinal imaging with hemoglobin A1c (HbA1c) and disease duration are widely applied [15–17]. For DN, models combining estimated glomerular filtration rate (eGFR) with blood pressure (BP) show robust accuracy [18–20], while CVD risk assessment using lipid profiles, such as total cholesterol (TC) and high-density lipoprotein (HDL), improves early detection [21]. Meanwhile, omics technologies provide novel insights: *APOL1* high-risk variants predict DN progression [22–24], metabolomics identifies DR-related signatures [25], and plasma symmetric dimethylarginine correlates with CVD risk [26]. Recent studies have increasingly explored the integration of multimodal data to leverage complementary information from multiple data sources in diabetic complications. For example, DeepOmix-FLEX combines proteomics, clinical data, and imaging for superior kidney disease prediction [27], while integrating metabolomics and peptidomics enhances DN diagnosis [28]. These advances suggest that cross-scale data fusion may become a new paradigm.

In this narrative review, we aim to summarize and compare AI-based predictive models for DR, DN, and CVD, focusing on models built from clinical, imaging, molecular omics, and multimodal data. The review covers 58 studies published over the past 10 years, between January 2015 and June 2025, identified through systematic searches of PubMed, Web of Science, and Scopus, including additional records from manual reference tracing and relevant reviews. We highlight the strengths and limitations of different modeling strategies, discuss multimodal integration approaches, and propose a six-step actional framework and clinical translation pathway for future research to support precision prevention and data-driven management of diabetic complications.

### Methods of literature search

A structured literature search was conducted to identify relevant studies applying AI for the prediction of diabetic complications, including DR, DN, and CVD. The databases PubMed, Web of Science, and Scopus were systematically searched for publications from January, 2015, to June, 2025. This 10-year timeframe was chosen to capture the modern era of AI and ML development in medical prediction models. A combination of keywords and Medical Subject Headings (MeSH) terms related to diabetic complications, AI and prediction models was used. The core search string structure was as follows: ‘diabetic retinopathy’ OR ‘diabetic nephropathy’ OR ‘diabetic cardiovascular disease’ AND ‘machine learning’ OR ‘deep learning’ AND ‘predictive model’ OR ‘early diagnosis.’ In addition to database searching, supplementary records were identified through manual reference tracing, Google Scholar, and screening of relevant reviews to ensure comprehensiveness. Studies were considered eligible for inclusion if they applied AI-based predictive models, including traditional ML, deep learning (DL), or large language models (LLMs), for DR, DN, or CVD. Eligible models utilized clinical variables, imaging data, molecular features, or combinations thereof. All retrieved records were imported into EndNote for duplicate removal, followed by a two-step screening process. Titles and abstracts were first reviewed to exclude irrelevant studies (e.g. non-diabetic complications, animal studies, or non-original works). Full-text evaluation was then performed according to the following exclusion criteria: (i) review articles or conference abstracts; (ii) studies lacking predictive modeling or AI application; (iii) studies without model validation; and (iv) studies not reporting any performance evaluation metrics, such as the area under the receiver operating characteristic curve (AUC), accuracy (ACC), or sensitivity (SN).

**Figure 1:**
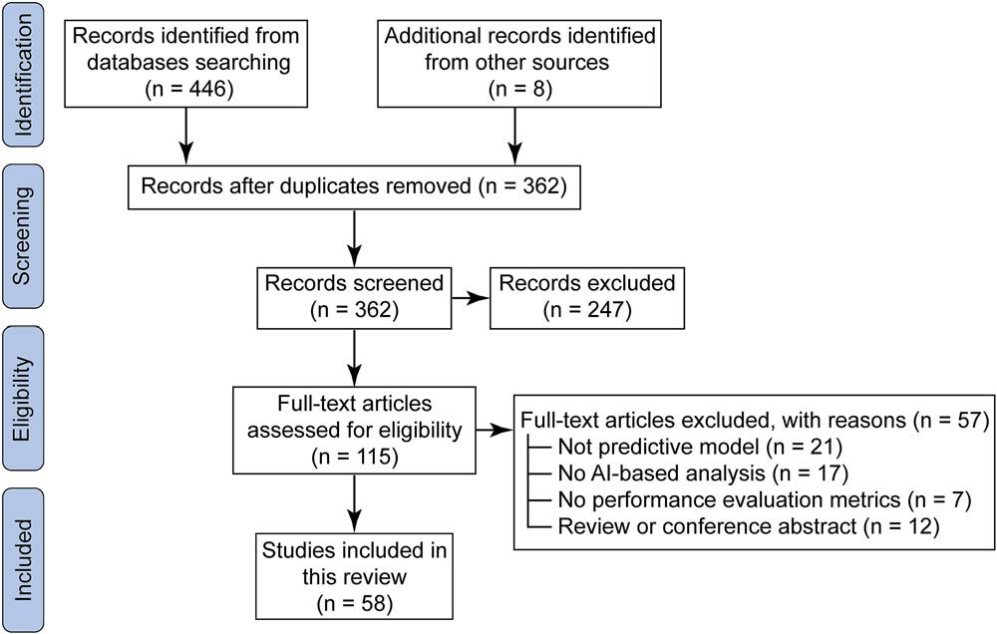
Flowchart of literature selection process. The flow diagram illustrates the identification, screening, eligibility, and inclusion steps of the literature search process. A comprehensive search was conducted in PubMed, Web of Science, and Scopus databases for studies published between January, 2015, and June, 2025, using the keywords: ‘diabetic retinopathy’ OR ‘diabetic nephropathy’ OR ‘diabetic cardiovascular disease’ AND ‘machine learning’ OR ‘deep learning’ AND ‘predictive model’ OR ‘early diagnosis.’ Additional records were identified through manual searches of relevant review articles and reference lists. After removing duplicates and excluding irrelevant, non-original, or incomplete studies, a total of 58 studies were finally included in this review.

A total of 446 records were identified through database searches, and eight additional records were obtained from other sources. After removing 92 duplicates, 362 records remained for title and abstract screening. Following the exclusion of 247 irrelevant studies, 115 full-text articles were assessed for eligibility. Finally, 58 studies meeting the inclusion criteria were included in this review. The entire process followed the Preferred Reporting Items for Systematic Reviews and Meta-Analyses (PRISMA) framework and is illustrated in Fig. 1.

## Diabetic retinopathy

**Figure 2:**
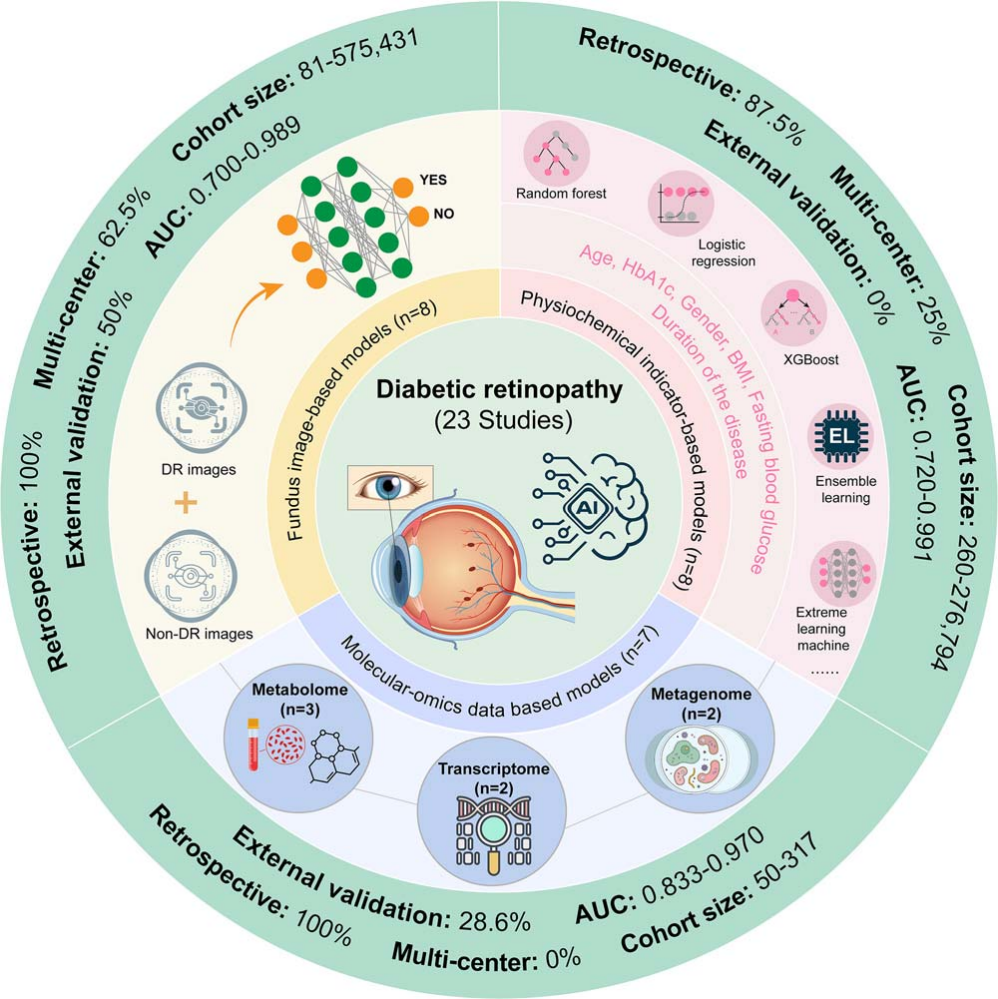
Overview of AI-driven predictive models for DR. Summary of 23 studies on AI-based DR prediction categorized by input modality: fundus images (*n* = 8 studies), clinical physiological features (*n* = 8), and molecular omics data (*n* = 7). Fundus image-based models commonly utilize DL techniques such as convolutional neural networks (CNN) to automatically detect retinal lesions. All (100%) were retrospective, 62.5% involved multicenter cohorts, and sample sizes ranged from 81 to 575 431 images. Half (50%) performed external validation, and AUCs ranged from 0.700 to 0.989. For clinical feature-based models, 87.5% were retrospective and 25% multicenter, including 260–276 794 participants. None performed external validation, with reported AUCs ranging from 0.720 to 0.991. For omics-based models, datasets included metabolomics (*n* = 3 studies), transcriptomics (*n* = 2), and metagenomics (*n* = 2). All (100%) were retrospective and single-center designed, with cohort sizes of 50–317 participants, and 28.6% had external validation. AUCs ranged from 0.833 to 0.970.

DR, one of the most prevalent microvascular complication of diabetes, is a leading global cause of vision impairment and blindness [5, 29–31]. It develops from chronic hyperglycemia-induced retinal microvascular damage, clinically categorized into non-proliferative (NPDR) and proliferative (PDR) [32]. With the escalating diabetes pandemic, DR prevalence continues to rise, affecting about one-third of patients during their lifetime and increasing risks for severe systemic complications such as stroke and CVD [33, 34]. Early screening and timely intervention are critical for preventing DR-induced blindness [32, 35, 36]. Current clinical diagnosis primarily relies on retinal vascular changes [37], while the rapid advancement of AI is driving new auxiliary diagnostic solutions (Fig. 2) [38].

### AI models for DR: clinical feature-based approaches

AI-based predictive modeling of DR using clinical data has primarily evolved along two major lines: image-based models that analyze fundus photographs, and physiochemical indicator-based models that utilize structured clinical and laboratory parameters. These two data modalities differ substantially in data scale, model architecture, and clinical applicability, yet both aim to enable early and accurate detection of DR risk (Table S1).

In total, 16 studies for clinical feature-based DR prediction were included in this review. Only one was prospective, while all others adopted a retrospective design. Among these, seven studies were multicenter, five of which were fundus image-based, suggesting that imaging models have been more frequently validated across sites. In contrast, physiochemical indicator-based models were predominantly single-center (75%, 6/8), with only 2 multicenter studies conducted in the United States and Northern Ireland [39, 40], reflecting a limited degree of external validation across populations. Most studies used retrospectively collected hospital or screening program data, whereas population-based screening cohorts or standardized follow-up periods were rarely reported. Sample sizes varied widely between modalities. Large-scale datasets were characteristic of image-based DL models, ranging from several thousand to over 500 000 retinal images, whereas physiochemical indicator-based models were typically limited to hundreds to tens of thousands of patients [41, 42]. This discrepancy is understandable, as each individual in imaging datasets may contribute multiple fundus images, thereby inflating the effective sample size. Regarding validation, almost all studies performed internal validation—by partitioning data into training and testing subsets or by cross-validation—while external validation, a critical indicator of generalizability, was conducted in only four studies (25%, 4/16). Population composition also differed notably. Multiethnic cohorts were common in fundus image-based studies, particularly in multicenter projects from the United States, Europe, and Asia, whereas physiochemical indicator-based models were largely derived from East Asian hospital cohorts without explicit demographic diversity assessment. Endpoint definitions varied considerably as well. Most imaging studies focused on referable or vision-threatening DR (moderate NPDR or worse) as the primary outcome, while others targeted diabetic macular edema or early microaneurysm detection. For clinical variable-based models, endpoints were typically based on ophthalmologist-confirmed diagnoses in hospital records or national databases. Moreover, the prevalence of DR across included datasets ranged widely, from 3% in screening cohorts to over 50% in hospital-based datasets, indicating potential class imbalance that may bias reported performance metrics.

Taken together, although most DR prediction models achieved impressive discriminative performance (AUC 0.70–0.99 for image-based and 0.72–0.99 for physiochemical models), while pronounced heterogeneity in study design, endpoint definition, and validation strategy hampers direct cross-study comparability and clinical generalizability. Furthermore, the paucity of prospective, multicenter, and externally validated studies limits the translational robustness of current DR risk prediction models. These findings underscore the urgent need for harmonized quality reporting and validation standards in future AI-driven DR prediction research. In the following sections, we summarize the methodological characteristics and recent advances of fundus image analysis models and physiochemical indicator-based models, respectively.

#### Fundus image analysis models

DL, particularly convolutional neural networks (CNNs), dominates AI applications in fundus imaging for DR prediction, enabling automated detection and grading to improve screening efficiency and reduce healthcare burden. For instance, large-scale studies using CNN architectures have achieved high accuracy in DR progression prediction, surpassing conventional risk factors, especially when combined with clinical data [41]. Models aligned with screening guidelines report AUC > 0.95 for referable DR in diverse populations [43]. The diagnostic capability of such systems has also been shown to be comparable to trained human graders in multi-ethnic settings [44].

However, reliance on standard fundus imaging revealed limitations. Traditional approaches for detecting complications like center-involved diabetic macular edema often required sophisticated and costly 3D imaging equipment, limiting accessibility, particularly in resource-constrained settings. Varadarajan et al. [45] developed a groundbreaking DL system that achieved accurate diagnosis using only widely available 2D fundus photographs, demonstrating performance comparable to gold-standard methods. Similarly, Bellemo et al. [46] pioneered the clinical validation of a DR screening model within an African screening program in Zambia. Their innovative ensemble DL architecture demonstrated exceptional performance in this real-world, resource-limited setting. These innovation highlighted AI’s potential to provide cost-effective screening solutions and revolutionize paradigms in diverse environments.

Basic CNN architectures sometimes struggled to focus effectively on the most discriminative lesions in complex retinal backgrounds. This limitation spurred the development of more sophisticated architectures incorporating attention mechanisms. Bhati et al. [47] developed IDANet, which innovatively integrates bidirectional spatial-channel parallel attention. This design allows the model to filter redundant information while precisely capturing DR-specific features like microaneurysms and exudates. Zhang et al. [48] employed multi-layer attention mechanisms within a deep neural network (DNN) to synergistically optimize both local lesion characteristics and global retinal status. Similarly, Ikram et al. [49] proposed a hybrid model that ingeniously fuses the local feature extraction strengths of CNNs with the global contextual understanding capabilities of Vision Transformers. This hybrid approach demonstrated superior performance over conventional CNNs or standard Transformers alone.

Collectively, these studies underscore substantial advancements in AI-powered DR prediction through fundus image analysis, driven by evolution from foundational CNNs to attention-enhanced and hybrid architectures. The field has progressed from demonstrating basic diagnostic capability to tackling challenges like data accessibility, geographical generalizability, and capturing intricate pathological patterns. Despite these impressive gains, challenge persist. Standardizing image quality variations and ensuring compatibility across different fundus camera devices remain unaddressed in current models. Furthermore, proof of cost-effectiveness compared to existing screening workflows is scarce. While adaptable to resource-limited settings, the economic benefit needs robust demonstration. Therefore, future research should prioritize: Developing lightweight, efficient models deployable on portable devices for point-of-care screening, and establishing unified standards for cross-device image preprocessing and feature alignment to handle real-world variability. Crucially, conducting clinical trials is imperative to validate the long-term clinical effectiveness and cost-efficiency of AI systems within real-world DR management ecosystems.

#### Physiochemical indicator-based models

Models based on physiochemical indicators predict DR risk using demographic data, laboratory tests, and medical history, offering interpretable and cost-effective solutions for clinical screening. Early studies demonstrated the utility of tree-based algorithms in capturing complex variable interactions. For example, a random forest (RF)-based model trained on 3000 diabetic cases achieved an AUC of 0.991, highlighting renal function, glycemic control, and inflammatory markers as key predictors [50]. Similarly, an Indian cohort study reported an RF-based model with AUC = 0.91 [42], underscoring the predictive strength of tree-based models.

To enhance predictive accuracy and scalability, recent studies adopted gradient boosting approaches and ensemble learning. For example, Liu et al. [51] developed an extreme learning machine (ELM) prediction model using electronic health records (EHR), outperforming traditional algorithms such as support vector machine (SVM), K-nearest neighbors (KNN), RF, and artificial neural network (ANN). A large-scale single-center analysis of 32,452 type 2 diabetes (T2D) patients implemented an eXtreme Gradient Boosting (XGBoost)-based model incorporating 17 physiological variables, including age, HbA1c, and fasting blood glucose, achieving superior reliability compared to other ML baselines [15]. Complementary findings were reported in a Romanian population-based study focusing on cardiovascular risk factors, where XGBoost confirmed BP, body mass index (BMI), and lipid levels as major predictors of DR progression [52].

Beyond conventional feature sets, adaptive and multi-task frameworks have emerged. A self-evolving XGBoost-based model demonstrated significant advantages in DR diagnosis, identifying gender, disease duration, and hypertension as critical predictors of DR risk [53]. Moreover, ensemble approaches have shown high accuracy in staging diabetic eye disease: distinguishing non-diabetic individuals, diabetes without DR, and DR with macular edema (accuracy = 0.84–1.00) using basic clinical and visual parameters [40]. Similarly, a U.S. study established a predictive model incorporating nine clinical variables including insulin use, age, and BMI, which effectively identifies high-risk ocular complications like PDR, referable retinopathy, and diabetic macular edema, providing decision support for screening prioritization [39].

Overall, tree-based algorithms, especially RF and XGBoost, dominate physiochemical-based DR prediction, consistently identifying renal function, long-term glycemic control, BP, lipid levels, and inflammation as major risk factors. However, most models are based on single-center or population-specific data, limiting generalizability. Additionally, model inputs are predominantly structured variables, while subjective and unstructured features such as lifestyle behaviors and functional assessments are underutilized. While traditional tree-based ML models remain dominant in DR prediction, the incorporation of advanced learning architectures such as DL or domain-adapted language models is recommended to improve generalization and clinical usability.

### AI models for DR: molecular-omics-driven approaches

Compared with studies based on clinical features, omics-based investigations of DR prediction remain relatively limited. In this review, seven studies were identified, including three metabolomics, two transcriptomics, and two metagenomics analyses (Table S2). All included studies adopted retrospective and single-center designs, with only two (2/7) employing untargeted approaches [54, 55], while the remaining primarily utilized targeted omics profiling. Unlike clinical feature-based models that typically leverage large-scale cohorts, omics studies were conducted on much smaller datasets, ranging from several dozen to a few hundred participants. This limitation largely reflects the high cost, technical complexity, and time-consuming nature of omics data generation, which constrain sample size expansion. Such limited sample sizes inevitably increase the risk of statistical overfitting, as complex omics features may lead to models that fit noise rather than true biological signals, thereby compromising reproducibility and external generalizability. Notably, most omics studies reported relatively balanced case–control ratios (~1:1) [56], contrasting with the frequent class imbalance seen in clinical datasets. This may result from deliberate sample matching or case–control balancing during experimental design. Regarding validation, only two studies utilized external validation based on public datasets, while the remaining five performed internal validation exclusively within their own cohorts. Regarding population composition, most cohorts were derived from Asian populations (primarily China and Turkey), while only a few transcriptomic studies utilized publicly available multi-regional datasets. Only studies using public resources provided accessible datasets, while none of the self-collected cohort studies shared raw data or model code.

Early studies demonstrated the potential of targeted metabolite profiling for disease staging. For instance, a hybrid AI model integrating SVM and multilayer perceptron distinguished non-DR, NPDR, and PDR stages in T2D patients with an accuracy of 89.58%, identifying serum biomarkers such as glucose, glycine, HbA1c, and creatinine as key determinants [57]. Building on this, XGBoost-based models incorporated explainable boosting techniques to refine metabolite-driven prediction, prioritizing six discriminative metabolites, including tryptophan, phosphatidylcholine diacyl C42:2, butyrylcarnitine, and dimethylarginine [58]. Additionally, targeted lipidomics combined with ML distinguished NDR from NPDR in type 1 diabetes (T1D) patients [56]. These metabolites not only enhance DR prediction but also provide mechanistic insight into disease progression.

Beyond metabolomics, transcriptomic profiling identified immune and oxidative stress-related signatures as pivotal in DR pathogenesis. A ML framework integrating seven algorithms identified novel immune-related genes that outperformed previous biomarkers in predictive accuracy [54]. Similarly, oxidative stress-related genes were prioritized through predictive modeling, achieving remarkable diagnostic performance [55]. These studies underscore the mechanistic depth offered by transcriptomics, providing candidate targets for future therapeutic development, although functional validation remains imperative.

Recent investigations have extended biomarker discovery to the gut microbiome, introducing an entirely non-invasive avenue for DR screening. A Chinese cohort using 16S rRNA sequencing found 25 discriminative bacterial families that accurately differentiated DR patients from diabetes and controls [59]. Another study reinforced these findings by identifying seven bacterial genera capable of distinguishing DR from healthy states with robust performance [60]. These findings suggest gut-retina axis perturbations may contribute to DR and offer opportunities for microbiota-based interventions.

Omics-driven models spanning metabolites, transcripts, and microbial profiles improve DR risk prediction and uncover mechanistic insights, moving beyond phenotypic screening toward precision diagnostics. Nevertheless, the targeted techniques deliver clinically translatable signatures yet restrict biomarker innovation through predefined analyte panels. Future research should emphasize the adoption of untargeted omics strategies to broaden the biomarker discovery space and uncover novel molecular signatures, accelerating de novo biomarker identification across ethnic cohorts, particularly for early NPDR where current signatures remain inadequate.

## Diabetic nephropathy

**Figure 3:**
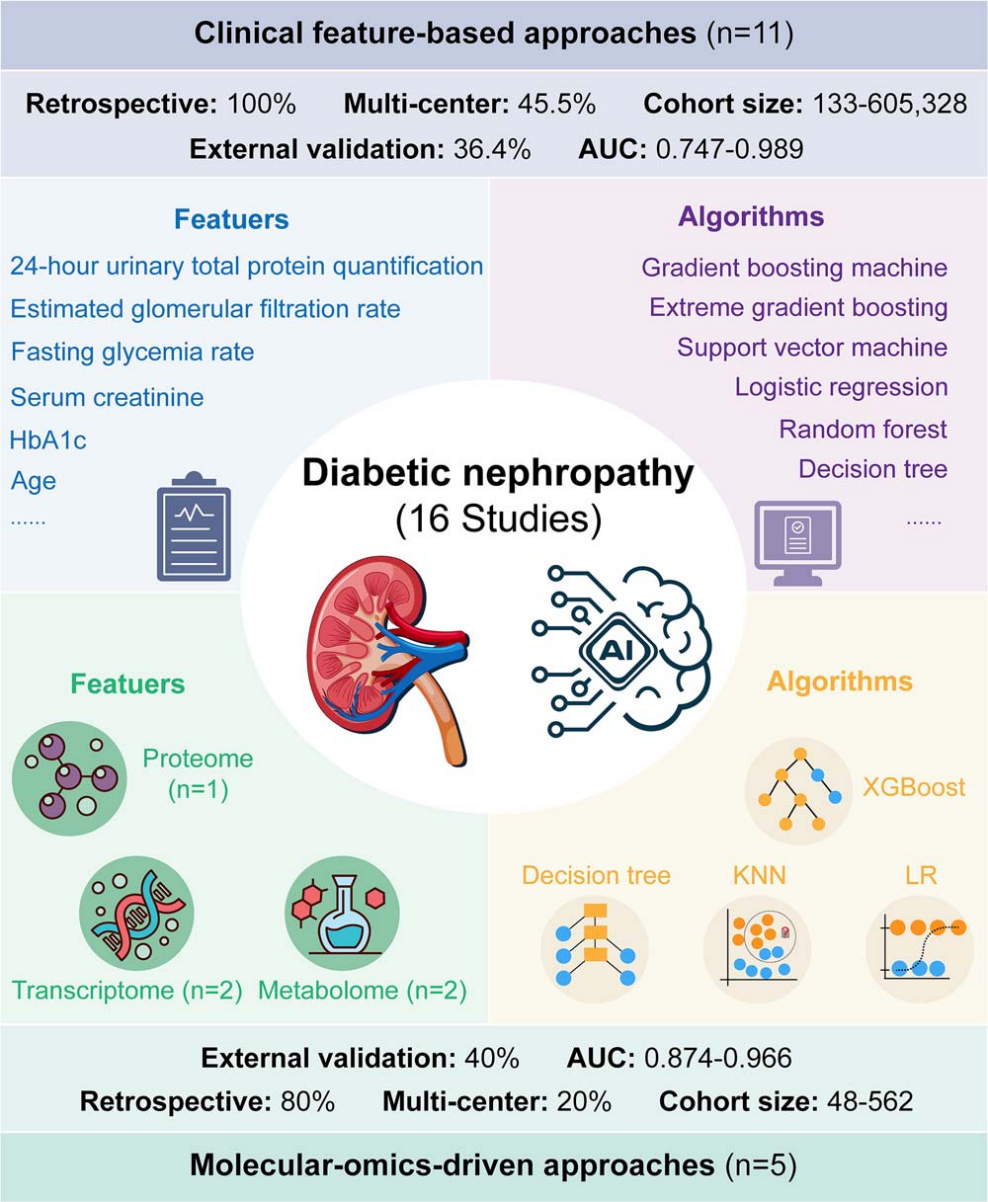
AI-powered predictive models for DN. A total of 16 studies on AI-based DN prediction were reviewed, including 11 using clinical feature-based models and five using molecular omics-based models. Among clinical feature-based models, all (100%) were retrospective, 45.5% were multicenter studies, and cohort sizes ranged from 133 to 605 328 participants. External validation was performed in 36.4% of studies, with reported AUCs ranging from 0.747 to 0.989. Clinical models typically incorporate variables such as renal function indicators and glycemic control measures, and commonly utilize traditional ML algorithms such as SVM and RF. For omics-based models, datasets included proteomics (*n* = 1 studies), transcriptomics (*n* = 2 studies), and metabolomics (*n* = 2 studies). Most (80%) were retrospective, 20% multicenter, with cohort sizes between 48 and 562 participants; 40% conducted external validation. Model performance (AUC) ranged from 0.874 to 0.966.

DN is a major serious chronic complications of diabetes, characterized by progressive renal dysfunction leading to ESKD [7]. DN usually begins with microalbuminuria and progresses to overt proteinuria with sustained renal decline, affecting 20%–40% of diabetic patients [61]. This condition not only accelerates renal damage but also forms a vicious cycle with cardiovascular events and retinopathy, profoundly impacting patients’ quality of life and clinical outcomes [62, 63]. Early diagnosis relies on persistent proteinuria monitoring and renal function assessment [64], while emerging biomarker detection and AI-powered diagnostic tools are actively being explored to enhance early detection capabilities and refine risk stratification strategies (Fig. 3).

### AI models for DN: clinical feature-based approaches

Similar to DR, clinical feature-based AI models for DN remain a primary focus of predictive modeling research. In this review, 11 studies employing clinical features for DN prediction were included (Table S3), all of which were retrospective and lacked prospective validation. Among these, ~45.5% (5/11) studies adopted multi-center designs. The geographical distribution of DN studies is broad, covering China, the USA, Iran, Singapore, Australia, and Northern Ireland, reflecting active global exploration in this field. Study populations predominantly comprised patients with T2D [65], with some studies also including T1D patients [66]. Prediction tasks were relatively focused, mainly aiming to forecast the occurrence of DN in diabetic populations. Endpoints were primarily defined based on proteinuria (urinary albumin-to-creatinine ratio ≥ 30 mg/g) and reduced glomerular filtration rate (eGFR <60 ml/min/1.73 m^2^) [67], while a few studies used renal biopsy pathology [68] or International Classification of Diseases (ICD) coding [69], indicating heterogeneity in endpoint definitions. Study sizes varied widely, ranging from several hundred participants in single-center cohorts to over 500 000 patients in large EHR databases. Multi-center studies typically relied on EHR systems or multinational screening programs, whereas single-center studies were mainly based on hospital inpatient or outpatient records. Regarding sample balance, DN incidence was ~30%–50%, indicating a generally balanced dataset. Notably, most studies did not specify the prediction horizon, with only a few targeting new-onset DN over 1- or 3-year periods [65, 70]. Regarding modeling approaches, tree-based models (e.g. RF, gradient boosting machines) and logistic regression (LR) were most commonly used, with some studies incorporating DL models to integrate fundus images with clinical variables. Common predictive features included HbA1c, serum creatinine, eGFR, diabetes duration, BP, and the presence of retinopathy or CVD history; these features were consistently identified as important determinants of DN onset. In terms of performance, reported AUCs ranged from 0.75 to 0.99, with most models achieving 0.80–0.90, indicating generally good discriminative ability. However, high performance was often based on internal validation, with external validation conducted in only 4 (4/11) studies, suggesting limited generalizability. Most studies did not provide code or data, with only a few based on public datasets offering partially reproducible resources, which restricts algorithm transparency and reproducibility.

Early predictive models primarily utilized structured data such as glycemic indices, renal function, and BP to identify high-risk individuals. For example, a study involving 210 Chinese T2D patients achieved an AUC of 0.850 using RF, emphasizing HbA1c, serum creatinine, and disease duration as key determinants for DN [67]. Similarly, a retrospective study employing decision trees (DTs) and genetic algorithms (GAs) highlighted the predictive value of age, HbA1c, systolic and diastolic blood pressure (SBP and DBP) [71]. A separate study involving 133 diabetic patients combined principal component analysis (PCA) with SVM, achieving 88.7% accuracy, demonstrating the effectiveness of feature selection combined with ML [72]. While these approaches demonstrated reasonable accuracy, their reliance on static variables and single-center cohorts limited generalizability and clinical adoption.

To improve predictive robustness, recent efforts have focused on leveraging large-scale EHR data and multicenter cohorts. Momenzadeh et al. [69] developed several complication prediction models, including DN risk stratification model with EHR data. Similarly, a time-enhanced gradient boosting machine (GBM) trained on EHR data from 14,039 patients with T2D achieved AUCs of 0.83, 0.78, and 0.82 for predicting DN onset over 2-, 3-, and 4-year horizons, underscoring the value of longitudinal data integration [70]. Another Iranian study used EHR data from 6235 T2D patients to build RF-based models with satisfactory predictive performance [73]. Zhu et al. [65] analyzed historical EHR data from 2184 Chinese T2D patients and developed an SVM-based model for DN prediction. Ravizza et al. [74] employed a data-driven feature selection strategy to identify seven key predictors, including age, BMI, eGFR, creatinine, glucose, albumin, and HbA1c, for chronic kidney disease (CKD) prediction with large-scale EHR data. Multicenter cohort data have also shown promise. For instance, a multicenter LR-based study identified diabetes duration ≥5 years, severe proteinuria, eGFR <30 ml/min/1.73 m^2^, and the presence of DR as independent predictors [68]. Du et al. [66] utilized a multicenter retrospective data to construct an XGBoost-based model for DN prediction. The model incorporated diabetes duration, postprandial glucose, SBP, HbA1c, serum creatinine, and low-density lipoprotein cholesterol (LDL-C), achieving robust cross-center prediction and supporting early risk assessment.

Beyond structured clinical data, emerging studies explore combining imaging biomarkers with demographic and biochemical parameters to enhance predictive performance in DN. For example, a DL model incorporating retinal fundus images alongside age and sex achieved AUCs of 0.866 internally and up to 0.828 in external validation cohorts, indicating the feasibility of cross-modality prediction for DN [75]. Such integrative approaches may provide surrogate indicators of renal microvascular health, particularly in settings where invasive renal assessment is impractical.

Overall, clinical feature-based AI models for DN prediction have evolved from early approaches utilizing limited structured data to more sophisticated models leveraging large-scale EHR and multicenter datasets. However, challenges such as inconsistent data coding, missing records, and variations in standardization associated with large-scale data integration continue to constrain model accuracy and generalizability. Traditional ML algorithms have demonstrated strong predictive performance. Feature selection techniques, including PCA and GA, further enhanced model accuracy, underscoring the importance of dimensionality reduction in handling complex clinical variables. Recent studies integrating longitudinal data through time-enhanced gradient boosting and expanding sample sizes across multiple centers represent a major step toward improving risk prediction. Furthermore, preliminary evidence supports the promise of DL for cross-modal frameworks, suggesting a future direction toward hybrid frameworks that integrate clinical, temporal, and imaging signals for early DN risk stratification.

### AI models for DN: molecular-omics-driven approaches

Compared with studies based on clinical features, research on DN prediction models driven by molecular-omics features remains relatively limited. This review included five relevant studies encompassing metabolomics, transcriptomics, and proteomics data (Table S4). The study designs were predominantly retrospective, with only one prospective cohort. Cohort sizes were generally small, ranging from 48 to 562 participants, and most studies were single-center; only two transcriptomics studies utilized public multi-cohort integrative to enable external validation [76, 77]. Similarly, in molecular feature-based DN models, the ratio of positive to negative cases was generally balanced, ~50% [78], helping to mitigate potential class imbalance effects on model performance. Regarding predictive features, metabolomics models mainly relied on serum small-molecule metabolites, such as amino acids and fatty acid derivatives [79]; transcriptomics models emphasized the expression of key genes related to oxidative stress and glomerular injury [76, 77], including *DUSP1, PRDX6, S100A8, PRKAR2B*, and *TGFBI*; proteomics models integrated plasma and urine protein expression information to predict risks of various kidney diseases, including DN. The employed algorithms were diverse, including XGBoost, DT, KNN, and LR. Two metabolomics studies used targeted experiments, while the remainder employed untargeted strategies. The reported performance was generally high, with AUC values ranging from 0.874 to 0.966, indicating strong discriminative capability of molecular-omics-based DN prediction models. Nevertheless, due to limited sample sizes, lack of external validation (2/5 have external validation), and restricted availability of data or code, the generalizability and reproducibility of these models remain constrained.

Integrating multi-omics data has markedly improved DN prediction and mechanistic understanding. Yin et al. [79] developed an XGBoost-based DN prediction model with serum metabolomics data, achieving an AUC of 0.966 and identifying eight potential metabolic biomarkers including acetylcarnitine, glutarylcarnitine, tyrosine, serine, and methionine. Similarly, another study involving 428 T2D patients applied ML to urinary and blood biomarkers, identifying features such as urinary albumin (uALB), uALB to creatinine ratio, cystatin C, and creatinine as strong predictors with DT [78]. Transcriptomics and oxidative stress-related markers have emerged as important dimensions for DN prediction. Using ML, one study identified three key oxidative stress-related genes, *DUSP1, PRDX6*, and *S100A8*, with strong diagnostic value [76], while Han et al. [77] highlighted *PRKAR2B* and *TGFBI* as potential markers for glomerular injury. Proteomics-based models further enabled early discrimination among CKD etiologies, suggesting utility for non-invasive DN diagnosis [80].

In summary, molecular-omics-driven AI models represent a transformative step in DN prediction, shifting the focus from conventional clinical indicators to molecular-level biomarkers that reveal early disease processes. Metabolomics has dominated current efforts, delivering high predictive accuracy and uncovering metabolic disruptions as early warning signals. Transcriptomic and oxidative stress signatures, along with proteomic profiles, provide complementary insights into pathogenic mechanisms and potential therapeutic targets. However, research on identifying molecular biomarkers associated with the heterogeneity of DN remains insufficient. Future studies should prioritize refined stratification of DN subtypes to better capture disease heterogeneity and guide precision interventions. In addition, the identification of biomarkers capable of predicting early renal function decline and trajectories of DN progression remains limited. Concurrently, the development of cross-scale dynamic monitoring technologies is essential to capture critical biological signals marking the transition from renal compensation to decompensation.

## Cardiovascular disease

**Figure 4:**
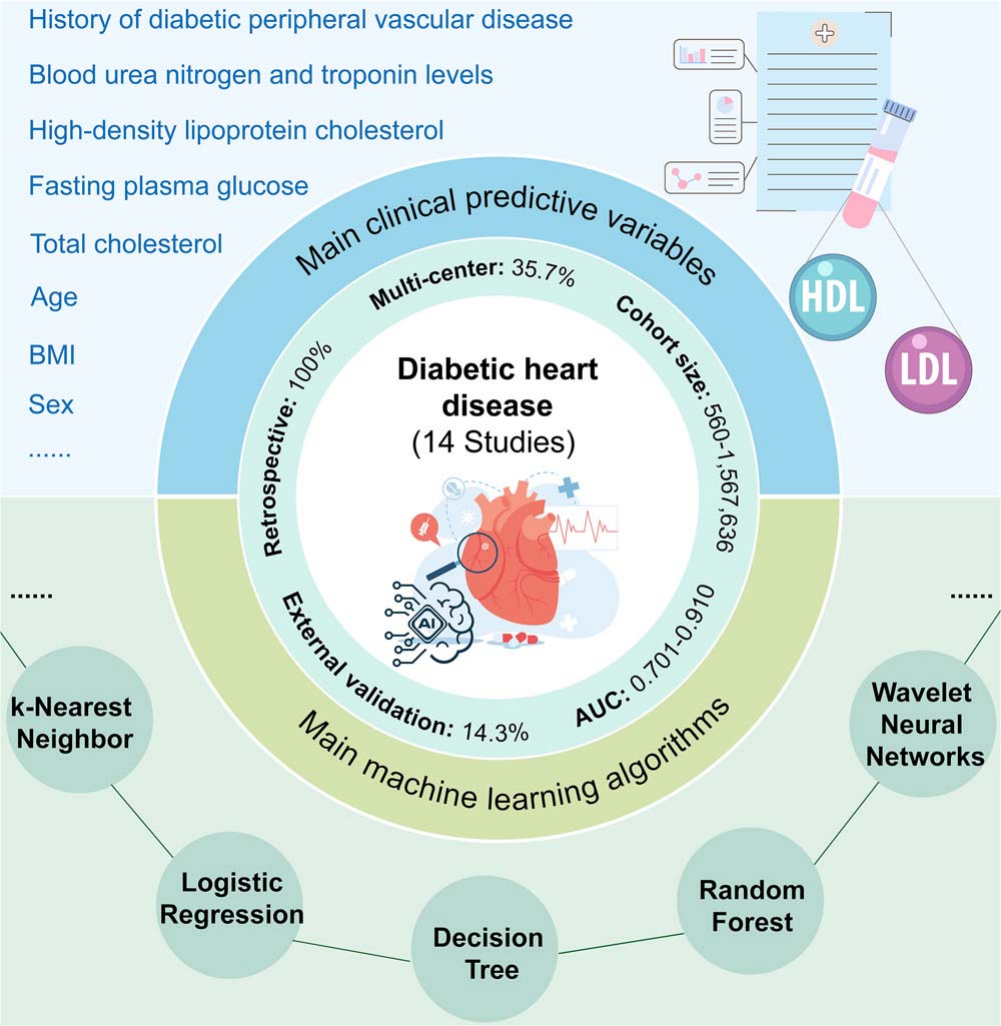
AI-based predictive models for CVD. A total of 14 studies on AI-based CVD prediction in diabetic populations were reviewed, all of which utilized clinical feature-driven models. All models (100%) were developed using retrospective cohorts, with 35.7% being multicenter studies. Cohort sizes ranged from 560 to 1 567 636 participants. External validation was performed in 14.3% of studies, and the reported model performance (AUC) varied from 0.701 to 0.910. These models typically incorporated structured clinical variables, such as age, sex, BMI, lipid profiles, and BP, and were built using both traditional ML algorithms (e.g. LR, DT, RF) and DL architectures. These models hold promise for early identification of high-risk individuals and precision prevention of CVD.

CVD, one of the most severe systemic complications of diabetes, remains the leading cause of mortality among diabetic patients [9, 81, 82]. Epidemiological studies reveal that diabetic patients face a 2- to 4-fold higher risk of developing CVD compared to the general population, with earlier onset and poorer prognosis [83, 84]. Early detection remains difficult due to the insidious nature of CVD and limited sensitivity of traditional diagnostics [85, 86]. Recently, AI has emerged as a promising tool in CVD diagnosis. By analyzing EHR, imaging data, and biomarkers, AI models demonstrate enhanced precision in risk prediction, early lesion identification, and even personalized treatment planning, thereby offering novel perspectives for clinical decision-making (Fig. 4).

### Clinical feature-based AI models

This review included 14 studies developing clinical feature-based AI models for CVD prediction (Table S5). All studies were retrospective, with single-center designs predominating, of which 64.3% (9/14) were single-center studies. The study populations spanned China, the USA, South Korea, Australia, Canada, Greece, and Bangladesh, reflecting the global interest in CVD risk prediction. Cohort sizes varied considerably, ranging from several hundred participants in single-center studies to over one million individuals in EHR databases. Multi-center studies typically relied on EHR systems or international collaborations [87, 88], while single-center studies were mainly drawn from hospital inpatient or outpatient records. The prediction tasks primarily focused on the occurrence or long-term risk of CVD in patients with diabetes, with some studies targeting specific cardiovascular events such as atherosclerotic CVD [89], major adverse cardiovascular events (MACEs) [90], coronary heart disease (CHD) [91], stroke, or heart failure [92]. Endpoint definitions were mainly based on clinical diagnosis, or ICD coding, with a few studies incorporating screening measures or biochemical criteria, indicating considerable heterogeneity in endpoints. Notably, approximately half of the studies explicitly specified a prediction horizon, most commonly ranging from 3 to 5 years, with 5-year predictions being particularly frequent, which holds clinical relevance for long-term risk management. Unlike DN studies with relatively balanced datasets, CVD prevalence ranged from 3% to 30%, and population balance varied across studies, potentially affecting model training and evaluation. Common predictive features included age, sex, BMI, BP, glycemic indicators (HbA1c, fasting blood glucose, FBG), lipid profiles (TC, HDL, LDL, triglycerides), renal function markers (creatinine, eGFR), diabetes duration, smoking history, and prior comorbidities, which were consistently identified as important determinants of CVD occurrence. Only two studies conducted independent external validation, while most relied on internal validation. Reported AUC values ranged from 0.701 to 0.910, with most models achieving 0.700–0.800; certain models using small single-center cohorts reached over 0.910. High performance typically came from internal validation, indicating that generalizability remains a challenge. Additionally, most studies did not provide data or code, limiting reproducibility and transparency.

AI-based models generally use demographic, clinical, and biochemical features to stratify CVD risk in diabetes. Early efforts primarily utilized smaller retrospective cohorts or survey-based data for CVD risk estimation. For instance, a study using retrospective cohort data applied the DT algorithm to predict comorbidity of diabetes and CVD, achieving an accuracy of 94.09% [93]. Similarly, an analysis compared eight ML algorithms, with RF performing best for early CVD detection [87].

Subsequent studies leveraged real-world EHR data to enhance predictive robustness. Lee et al. [92] developed a model using South Korean EHR data to predict cardiovascular complications in newly diagnosed T2D patients, emphasizing the practicality of models built on readily available clinical variables. Likewise, Ravaut et al. [88] applied ML to Canadian administrative health data, achieving satisfactory performance for adverse CVD events. In China, an RF-based model trained on demographic and laboratory data predicted 3-year CVD risk among T2D patients with robust internal and external validation [89]. However, limited prediction horizons and geographic homogeneity remain notable limitations.

Building on these efforts, larger cohorts and more advanced algorithms have been employed to improve performance and interpretability. A study involving 9059 participants developed an XGBoost model for MACE prediction in T2D, attaining an AUC of 0.82 and identifying phosphate and blood urea nitrogen as novel predictors [90]. Similarly, Athanasiou et al. [94] analyzed 5-year follow-up data of Greek T2D patients using XGBoost, producing interpretable personalized risk estimates for fatal and non-fatal CVD events. Zarkogianni et al. [95] further explored hybrid neural network architectures. At a larger scale, Ma et al. [91] constructed an XGBoost-based comorbidity model for diabetes and CHD using data from 300,000 patients in southwest China, identifying key metabolic and behavioral risk factors.

The integration of neural networks and behavioral determinants represents another emerging trend. An artificial neural network-based model achieved 92.86% accuracy to predict CVD risk in patients with diabetes [96]. Chu et al. [97] further incorporated psychosocial determinants into a DNN framework, achieving AUC = 0.91, suggesting that psychosocial and behavioral variables provide additional predictive value beyond traditional biomarkers. Hossain et al. [98] combined network feature analysis with ML to develop a novel prediction model for CVD risk in T2D patients, with an accuracy of 87.50%. Miao et al. [99] used SVM and KNN algorithms to predict CVD risk. Furthermore, a Korean cohort constructed an RF-based model, with Creatinine and HbA1c were identified as the most influential features [100].

In conclusion, clinical feature-based AI models for CVD risk prediction in diabetes have evolved from early, small-scale studies to large cohort-based approaches leveraging advanced algorithms. Tree-based models, particularly RF and XGBoost, dominate the field due to their ability to capture complex nonlinear interactions. However, limitations remain. Despite promising performance in models incorporating psychosocial factors, most CVD prediction frameworks still overemphasize traditional metabolic parameters, with insufficient exploration of key drivers such as psychological and behavioral traits, inflammatory biomarkers, environmental exposures, and lifestyle factors. This narrow feature space constrains interpretability and limits the translation of model outputs into actionable interventions. Furthermore, current models largely rely on static, cross-sectional clinical indicators, overlooking the long-term dynamic characteristics and intricate temporal dependencies underlying CVD progression. Such a ‘snapshot-based’ approach fails to capture critical signals such as glycemic variability, BP fluctuations, and drug-response trajectories. Additionally, most studies adopt passive learning paradigms, lacking dynamic risk modeling informed by causal inference and reinforcement learning, which is essential for real-time optimization of individualized treatment strategies. Future research should transcend the conventional ‘static features + classifier’ paradigm and advance toward causality-driven, multimodal predictive systems.

## Multimodal AI modeling strategies for diabetic complications

Researchers have increasingly investigated multimodal data integration strategies to leverage complementary information from different data sources for diabetic complications prediction, enabling a more holistic representation of disease biology and patient heterogeneity. A summary of representative multimodal studies is presented in Table S6. In total, five multimodal modeling studies were reviewed, covering all three major complications, with most focused on DN (*n* = 3) and CVD (*n* = 2), while DR was represented by a single study. All included studies adopted feature-level fusion approaches, in which different features were jointly incorporated into a unified model. The integrated modalities typically combined traditional clinical parameters (e.g. demographic and metabolic indices) with molecular omics data, including metabolomics and proteomics, with metabolomics being more prevalent (4 of 5 studies). Compared with the unimodal studies that were predominantly retrospective and single-center in design, the multimodal investigations displayed a relatively higher proportion of prospective (3/5) and multicenter (3/5) studies.

Importantly, these studies consistently demonstrated that integrating omics information into clinical models substantially enhanced predictive performance. For instance, in the Asian cohort by Sabanayagam et al. [101], combining serum metabolomic features with clinical covariates improved AUC from 0.797 to 0.843 for DN prediction. He et al. [102] demonstrated that incorporating metabolic features into traditional clinical models improved the prediction of both DR and DN, yielding higher AUCs in both internal (0.838 versus 0.743 for DN; 0.790 versus 0.764 for DR) and external validation (0.791 versus 0.691 for DN; 0.778 versus 0.760 for DR). Huang et al. [103] reported that a multimodal model combining two metabolites, sphingomyelin C18:1 and phosphatidylcholine diacyl C38:0, with clinical variables achieved optimal prediction of DN, yielding an AUC of 0.825, which outperformed the model based solely on established clinical predictors (AUC = 0.800). For CVD, multimodal strategies combining proteomic or metabolomic markers with clinical features also showed improvement in predictive accuracy. Shen et al. [104] reported that a metabolite-assisted model incorporating four key metabolites and nine clinical factors achieved an AUC of 0.962–0.979, significantly outperforming clinical-only models (AUC 0.576–0.745). Moreover, metabolic biomarker-assisted models showed higher net benefits than clinical-only base models across a wide range of risk thresholds, indicating improved clinical utility. Likewise, Looker et al. [105] demonstrated that the inclusion of six circulating protein biomarkers increased the AUC from 0.66 to 0.72, with a net reclassification improvement (NRI) of 8.1%.

Overall, Multimodal data has proven advantageous for complication prediction, yet notable limitations persist. The included studies mainly employed feature-level fusion, while decision-level or representation-level integration frameworks remain rare. Moreover, external validation and standardized benchmarking are limited, impeding cross-study comparability. None of the included studies performed comprehensive clinical cost–benefit analysis, and only one reported clinical utility evaluations using decision-curve analysis (DCA) [104]. Future studies should systematically evaluate the clinical utility of multimodal AI systems, including both decision-analytic approaches (e.g. DCA) and health economic evaluations (e.g. cost-utility and cost-effectiveness analyses), while also assessing predictive performance metrics such as calibration to ensure robust and reliable translation into clinical practice.

## Development of AI-based diagnostic models for diabetic complications

**Figure 5:**
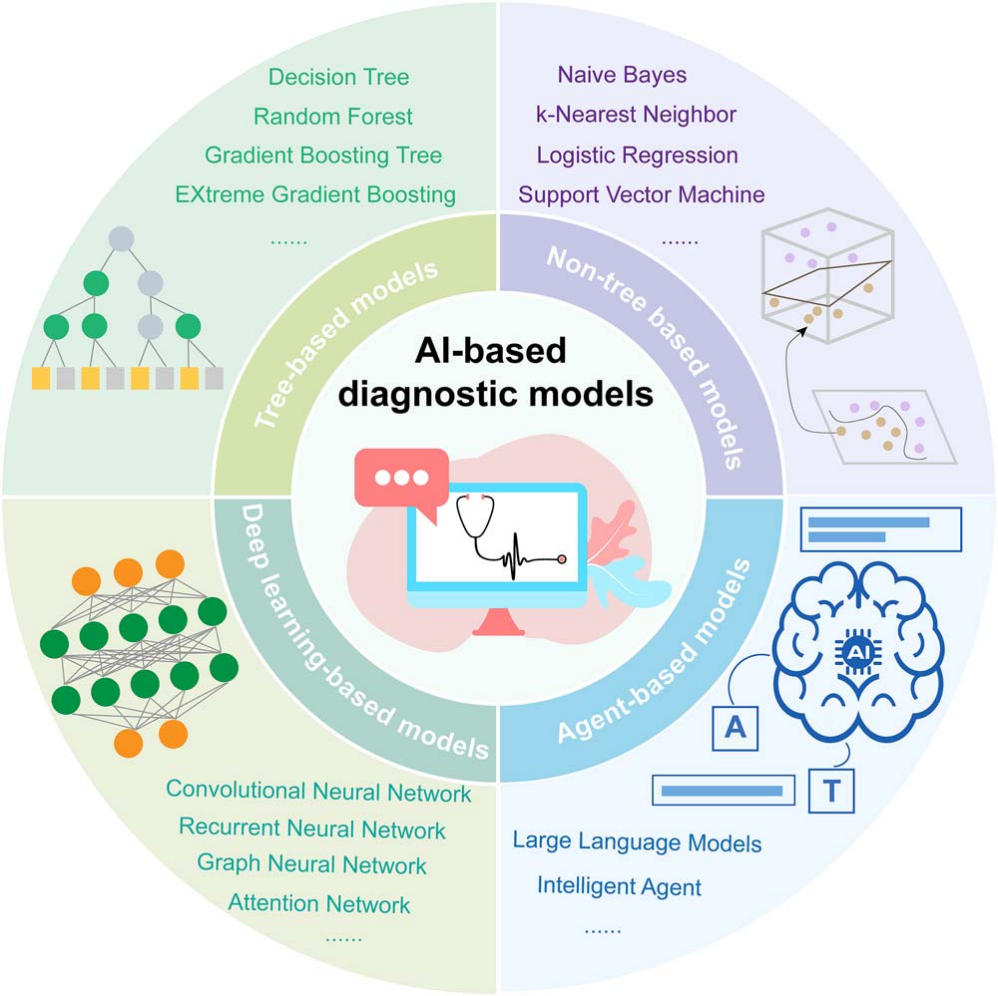
Overview of AI algorithm categories applied in diabetic complication prediction. AI algorithms employed in diabetic complication modeling can be broadly grouped into three major categories reflecting their methodological principles and evolutionary stages. Traditional ML algorithms include both tree-based models (e.g. RF, XGBoost) and non-tree models (e.g. SVM, LR), which rely on handcrafted feature inputs and structured data. DL algorithms, such as CNN, automatically learn hierarchical feature representations from high-dimensional data like medical images and multi-omics profiles, offering superior capability for nonlinear pattern recognition. Emerging LLMs and agent-based frameworks represent a new paradigm that integrates multimodal data understanding, reasoning, and clinical decision support. Together, these algorithmic categories constitute the technological backbone of AI-driven predictive modeling for diabetic complications, each contributing distinct strengths in feature extraction, interpretability, and scalability.

AI has played a significant role in the development of diagnostic models for diabetic complications, driving the evolution of predictive approaches from relying on single clinical features to integrating multimodal data. To address different data characteristics and clinical application needs, researchers have extensively explored various AI algorithms, including traditional ML methods, DL techniques, and emerging LLMs and agent-based technologies (Fig. 5). These algorithms differ not only in their technical principles but also fundamentally in their capabilities for feature extraction and pattern recognition.

### Traditional machine learning algorithms

Traditional ML algorithms have been the cornerstone of early AI-assisted diagnostic models [106–110], particularly demonstrating robust performance in scenarios where features are well defined and sample sizes are moderate. The core process typically includes feature engineering and model training/classification based on these features [111]. According to model structure and principle, these methods can be broadly categorized into tree-based models and non-tree-based models.

#### Tree-based models

Tree-based algorithms are preferred in clinical applications due to their strong feature selection capability, robustness to missing data, and high predictive performance. Common tree-based models include DT, RF, GBM, and XGBoost, all excelling in handling nonlinear relationships and multivariable interactions across DR, DN, and CVD.

DT construct a tree-like structure by recursively splitting the dataset into smaller, more homogeneous subsets based on criteria such as information gain or Gini impurity [112]. DTs are simple, interpretable, and require minimal preprocessing. Afrash et al. [71] demonstrated that a DT-based clinical feature model achieved superior discriminatory performance in predicting DN progression risk, while Gao et al. [78] applied DT to serum metabolites for DN prediction (AUC = 0.874). However, DTs are prone to overfitting and sensitive to data perturbations, limiting generalizability [113]. A single DT can also become overly complex, improving training accuracy at the expense of model simplicity and robustness.

To overcome the limitations of a single tree, ensemble learning methods have been widely applied. By combining multiple weak learners (typically DT), these approaches build a strong learner that significantly improves model generalizability and stability. RF is a classic example: it uses DT as base learners and aggregates the predictions of multiple independent trees to produce the final decision [114]. RF can effectively handle high-dimensional data while offering high predictive accuracy and strong parallel computing capability [115]. Importantly, RF naturally provides feature importance measures, which helps identify the most critical features for predicting complication risks. For example, Shen et al. [104] used a RF-based model to evaluate CVD risk in diabetic patients assisted by metabolic features, selecting metabolic biomarkers with a mean decrease in Gini >1.0 for model construction and training.

Among ensemble learning methods, gradient boosting trees and its enhanced variant XGBoost have been widely used in diabetic complication risk prediction due to their excellent performance and flexibility [116, 117]. XGBoost incrementally builds new trees to correct the residuals of previous predictions, integrating multiple weak learners into a strong learner. In addition, XGBoost supports automatic feature selection, missing value handling, and parallel computation, greatly enhancing training efficiency, especially suited for large-scale, high-dimensional clinical and omics data modeling. In AI-based studies of diabetes complications, XGBoost is one of the most commonly used ML classifiers. For instance, Li et al. [15] built a DR risk prediction model with XGBoost, identifying key features such as age, FBG, and HbA1c. Chu et al. [97] applied XGBoost framework with psychosocial variables, significantly improving CVD risk prediction. Despite its advantages in accuracy, XGBoost has a more complex model structure than DT, with numerous hyperparameters requiring careful tuning for optimal performance. Moreover, its interpretability remains relatively limited, and translating model outputs into concise, user-friendly clinical decision support continues to be a major challenge for future research.

#### Non-tree-based models

Non-tree-based algorithms have also played an important role in AI modeling for diabetic complications. These algorithms include LR [118], SVM [119], and KNN [120]. They are widely applied in clinical feature data modeling due to their solid theoretical foundations, high computational efficiency, and ease of interpretation. Non-tree-based models are particularly suitable for small to medium-sized datasets or scenarios with relatively limited feature dimensions. For example, Zhu et al. [65] developed a SVM-based risk prediction model for DN complications based on real-world EHR data from China. Similarly, Miao et al. [99] used SVM and KNN algorithms to develop a CVD risk prediction model for T2D patients. Both algorithms demonstrated high accuracy and recall, confirming the feasibility and value of non-tree-based models in specific contexts. However, due to their limitations in handling high-dimensional data and complex variable interactions, recent studies have increasingly focused on ensemble learning and DL methods to further enhance predictive performance.

### Deep learning

As a major branch of AI, DL builds neural networks with multiple layers of nonlinear mappings, enabling automatic learning of complex feature representations directly from raw data without relying on handcrafted features [121–123]. Compared with traditional ML, DL is particularly effective at capturing nonlinear relationships and latent feature structures within data.

In AI modeling of diabetic complications, DL techniques have been widely applied to the automated analysis of medical imaging data, with outstanding performance in the prediction and grading of DR [124, 125]. Deep CNN-based models have become the mainstream approach for automated interpretation of fundus photographs. These models can directly extract multi-level features from images, automatically detect lesions such as microaneurysms and hemorrhages, and effectively support early screening and grading of DR. For example, Li et al. [43] developed a deep CNN-based DR screening system that achieved performance comparable to or exceeding that of professional ophthalmologists across real-world datasets from multiple countries. In addition, DL models have gradually been applied to other diabetic complication prediction tasks, including the development of multimodal neural networks that integrate clinical variables, omics features, or time-series data to enhance risk prediction for DN and diabetic cardiovascular complications [126–128].

The strengths of DL lie in its powerful feature learning capabilities, elimination of manual feature engineering, ability to model complex nonlinear relationships, and good generalization performance on large datasets. However, there are also some limitations, such as heavy reliance on large-scale labeled data, high computational resource requirements during training, complex training and tuning processes, and relatively weak interpretability. These factors limit the large-scale deployment of DL models in real clinical environments to some extent. Therefore, enhancing model transparency, reducing dependence on labeled data, and developing lightweight, deployable DL architectures are important directions for future research.

### Large language models and agents

The integration of LLMs and their agent frameworks has emerged as a research hotspot in medical AI, showing considerable potential in the intelligent diagnosis and management of diabetes-related complications. In DR detection, recent advancements in foundation models have demonstrated strong transferability and generalization across diverse disease prediction tasks. For instance, the RETFound model [129] was pretrained on over 1.6 million unlabeled retinal images using self-supervised learning, enabling efficient adaptation to multiple downstream tasks, including the diagnosis and prognosis of vision-threatening eye diseases such as DR. Ayhan et al. [130] employed the multimodal Gemini 1.5 Pro model with contextual learning to perform binary DR classification, achieving an accuracy of 0.841 (95% CI: 0.803–0.879) and an F1 score of 0.88 (95% CI: 0.844–0.909), comparable to RETFound, without requiring retraining or large-scale labeled datasets. Similarly, Chetla et al. [131] utilized ChatGPT-4 Omni to grade DR severity on the EyePACS dataset, achieving high precision for PDR detection (accuracy 75.6%, precision 92.2%), demonstrating the feasibility of general-purpose LLMs for low-cost DR screening. Beyond image classification, Gopalakrishnan et al. [132] used LLMs to generate DR screening recommendations based on synthetic patient scenarios, showing that the models could simulate clinician reasoning in risk stratification. Mohammadi and Nguyen [133] combined ChatGPT with Vertex AI to implement programming-free retinal image preprocessing and ML training, yielding strong performance for both DR detection and severity assessment (AUC = 0.90 and 0.81). Moreover, Aftab et al. [134] reported that ChatGPT-4 exhibited high sensitivity for referable DR, reaching 96.2% in high-risk populations, but its limited specificity and tendency to overcall disease reduce its practicality as a general screening tool, while supporting its use as an auxiliary triage system for high-risk patients. Li et al. [135] developed DeepDR-LLM, a multimodal vision-language system for DR diagnosis and management, enabling multimodal generation of diagnostic and treatment recommendations and providing support for personalized diabetes care.

In renal function research, Bobart et al. [136] developed a biomedical BERT-based natural language processing model to automatically extract diagnostic information from kidney biopsy reports, achieving an average AUC of 0.95 across reports for DN, lupus nephritis, and other diseases, reducing manual annotation workload and demonstrating the scalability and accuracy of LLMs for pathological report structuring and clinical data mining. Cheng et al. [137] used ERNIE Bot 4.0, GPT-4o, and ChatGLM4 to generate patient education materials for early DN, and comparisons with physician-authored texts showed that LLM outputs were comparable or superior in accuracy, completeness, and readability, highlighting their potential in patient education and health management. For cardiovascular applications, Lorenzoni et al. [138] employed ChatGPT-4o to generate primary and secondary prevention information for myocardial infarction, with cardiology experts evaluating accuracy and readability. The model provided accurate and comprehensive answers for 75% of items, though readability was limited for highly specialized clinical topics, underscoring the need for professional oversight and validation in clinical deployment. Overall, LLM applications in diabetes-related complication management are expanding from data structuring and clinical text understanding to intelligent interaction and personalized patient education, offering new technological pathways for building intelligent systems for diabetes complication diagnosis and care.

Despite the promising potential of LLMs in the detection and management of diabetes-related complications, their large-scale deployment in real-world clinical settings remains constrained by several challenges. First, factuality bias and hallucination are among the most critical limitations [139]. Since LLMs are primarily trained on general-purpose corpora rather than rigorously validated medical knowledge bases, their outputs may contain inaccurate or even fabricated information, which could have serious consequences in disease risk assessment and treatment recommendation scenarios. Second, although the generated text is often linguistically fluent, it typically lacks clear reasoning interpretability and uncertainty quantification (UQ) mechanisms, making it difficult for clinicians to assess the reliability of outputs. Additionally, LLMs exhibit significant domain generalization issues: general-purpose models often show uneven performance across medical tasks. For instance, in DR grading tasks, ChatGPT-4 Omni performs well for PDR detection but shows markedly lower accuracy for mild or early-stage lesions [131], highlighting insufficient robustness under domain shift [140]. Finally, data security and patient privacy constitute another major challenge. Medical data are highly sensitive, and cloud-based deployment and inference of current LLMs increase the risk of potential privacy breaches.

To address these challenges, studies have proposed several strategies tailored to medical applications. First, Retrieval-Augmented Generation (RAG) has emerged as an effective paradigm [141]. By dynamically retrieving authoritative medical literature, clinical guidelines, or EHR knowledge bases before text generation, RAG provides external, verifiable knowledge support, thereby significantly reducing hallucinations and factual errors. For instance, Zhu et al. [142] developed the HaluEval-Wild benchmark based on RAG to evaluate hallucination rates in LLMs. Their study showed that GPT-4 exhibited a 20% hallucination rate without RAG, which dropped to 5% when RAG was incorporated. Expert feedback further indicated that RAG-enhanced GPT-4 not only reduced hallucinations but also offered comprehensive contextual information, thorough consideration of parameter settings, and clear explanations of underlying assumptions. Second, the introduction of UQ and confidence calibration techniques enables models to provide confidence scores or intervals alongside their outputs, allowing clinicians to make risk-stratified, human-in-the-loop decisions [143]. To address domain shift, researchers have proposed domain adaptation and few-shot fine-tuning strategies. For example, Ding et al. [144] introduced the 3DS (Decomposed Difficulty-based Data Selection) framework, which adopts a model-centric approach to align data selection with the model’s knowledge distribution, thereby improving adaptability and robustness in medical tasks. Additionally, parameter-efficient fine-tuning (PEFT) using limited labeled data can substantially enhance LLM performance under specific patient populations or device conditions, facilitating diverse downstream applications [145]. Overall, the synergistic integration of RAG, UQ, and domain adaptation strategies provides a feasible pathway to improve the usability, safety, and transparency of LLMs in medical contexts. In the management of diabetic complications, LLMs and their agents are particularly suited for integrating multimodal information, providing auxiliary personalized clinical decision support, and facilitating continuous patient engagement through conversational interaction.

As LLMs increasingly transition into clinical decision support systems, establishing robust evaluation standards and regulatory frameworks has become a critical challenge. Traditional natural language processing metrics, such as Bilingual Evaluation Understudy (BLEU) or Recall-Oriented Understudy for Gisting Evaluation (ROUGE), fail to fully capture the practical value of medical applications, highlighting the need for multidimensional evaluation criteria encompassing factuality, safety, and clinical reproducibility. For instance, in diabetes complication tasks, including DR, DN, and CVD, assessment should go beyond predictive accuracy to consider the medical soundness of outputs in patient stratification, treatment recommendations, and health education content. Wang et al. [146] proposed the Clinical Safety-Effectiveness Dual-Track Benchmark (CSEDB), designed by 32 specialist physicians, covering 26 clinical departments and 30 key indicators, including critical illness recognition, medication safety, and guideline adherence. Their study demonstrated an average 13.3% performance drop in high-risk scenarios, with general-purpose models underperforming domain-specific medical LLMs in both safety and effectiveness metrics. Similarly, Asgari et al. [147] developed a framework to evaluate hallucination and clinical safety in medical text summarization, showing that through optimized prompts and workflows, LLMs could reduce critical error rates below human note-taking levels, providing a concrete example of safe clinical documentation automation. From a regulatory and legal perspective, deploying medical LLMs requires clear responsibility allocation, clinician oversight mechanisms, and patient safety safeguards. For instance, when model outputs include diagnostic or therapeutic recommendations, it is essential to establish traceable decision pathways, record model inputs and outputs, and ensure that critical clinical decisions are reviewed by qualified professionals. Moreover, development and deployment should adhere to data minimization and de-identification principles, with transparency achieved through model cards and data cards. Integrating regulatory sandboxes and international standards can provide actionable pathways for review and validation of novel medical LLMs. These measures not only help identify potential regulatory risks, such as hallucinations, domain shifts, or inappropriate treatment suggestions, but also support the safe integration and continuous optimization of models in real-world clinical settings. Only within a framework that combines scientific validation, ethical compliance, and policy coordination can LLMs safely transition from research prototypes to clinical practice, advancing intelligent management of diabetes complications toward explainable, regulatable, and sustainable care. In conclusion, when generating medical advice, LLMs should be positioned strictly as assistive tools rather than autonomous decision-makers, and must operate within a controlled and auditable framework.

## Actionable framework and clinical translation pathway of AI in diabetic complications

**Figure 6:**
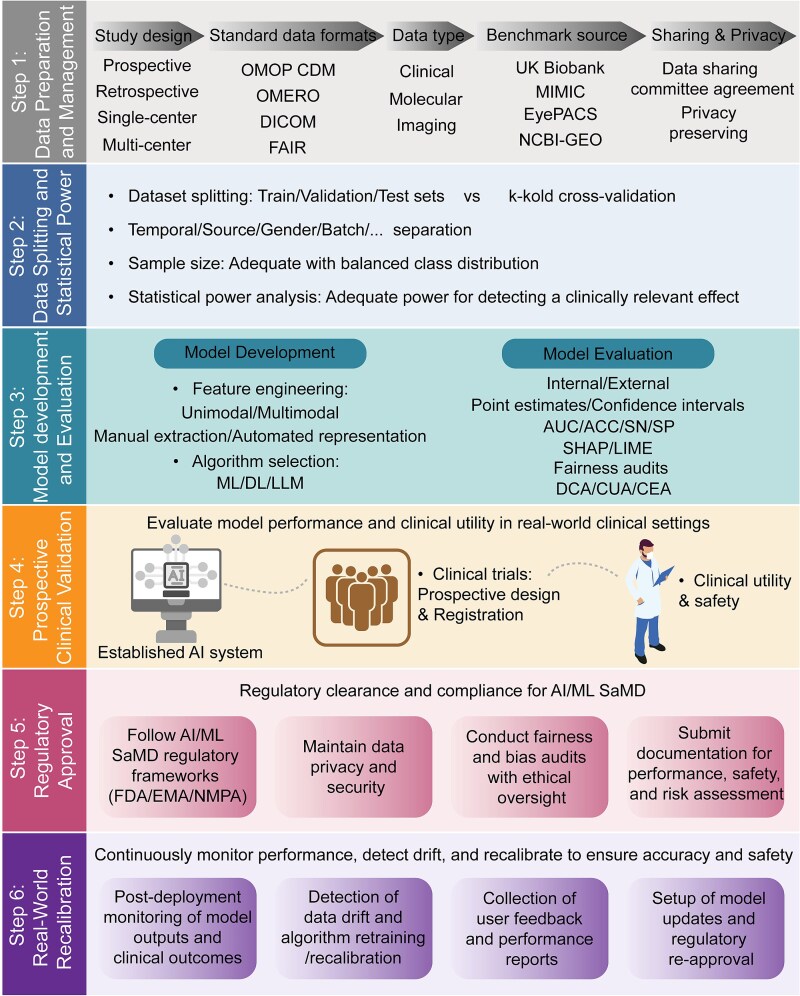
Integrated framework and translational roadmap of AI for predicting diabetic complications. The complete pipeline of AI model development encompasses six key stages. The framework begins with data preparation and management, emphasizing standardized data formats, benchmark datasets, and privacy-preserving data sharing. The data partitioning and statistical power stage focuses on rigorous dataset splitting strategies and adequate sample size. Model development and evaluation cover the entire process from feature engineering and algorithm selection to internal and external validation. The prospective clinical validation stage is crucial, testing model performance and utility in real-world clinical settings. Upon successful validation, the regulatory approval stage follows, ensuring compliance with frameworks established by FDA, EMA, and NMPA for market clearance. Finally, the real-world recalibration stage maintains model performance and safety through continuous monitoring and iterative updates after deployment.

Although AI has demonstrated great potential in predicting diabetic complications, the pathway from research prototype to a clinically reliable tool remains full of challenges. To address these challenges, based on the previous review, we propose a systematic and actionable six-stage framework for AI model development and clinical implementation (Fig. 6). This framework encompasses the full translation pathway, from data preparation, model development, and clinical validation to regulatory approval and real-world recalibration, providing practical reference for researchers, clinicians, and policymakers.

A robust AI model begins with high-quality, standardized data. During the study design phase, the research type (e.g. retrospective versus prospective, single-center versus multi-center) should be clearly defined, and internationally recognized data standards and sharing principles should be strictly followed, including the OMOP CDM (Observational Medical Outcomes Partnership Common Data Model), OMERO (Open Microscopy Environment Remote Objects), DICOM (Digital Imaging and Communications in Medicine) standards for medical imaging, and the FAIR (Findable, Accessible, Interoperable, Reusable) data principles. The data typically encompass clinical indicators, molecular omics profiles, and medical imaging information. We encourage the use of standardized benchmark datasets (e.g. UK Biobank, MIMIC, EyePACS, NCBI-GEO) to enhance reproducibility and cross-cohort comparability. Meanwhile, responsible data sharing should be promoted through controlled mechanisms such as data-sharing committee agreements and privacy-preserving technologies to ensure patient confidentiality.

In the stage of data splitting and statistical power analysis, rigorous methodology is crucial to ensure unbiased and reliable model evaluation. Datasets should be properly divided into training, validation, and test sets, and stratified by time, source, or batch where necessary to prevent information leakage. For smaller datasets, cross-validation or leave-one-out methods can be employed. In addition, sufficient sample size and balanced class distribution should be ensured, and statistical power analysis should be performed at the study design stage to confirm adequate power to detect clinically meaningful effects.

During the model development and evaluation stage, the core objective is to construct a high-performance and trustworthy model. Feature engineering and algorithm selection are two key steps during model development. Features may be derived from single- or multi-modal data, with extraction methods categorized as manually designed or automatically learned. Algorithm selection should align with data characteristics and task objectives, ranging from conventional ML and DL to LLMs. Model evaluation should include both internal and external validation. Internal validation assesses performance on an independent test subset, while external validation tests generalizability on a newly acquired cohort. External validation is the gold standard for assessing model generalizability, yet it is seldom implemented in current AI research. We therefore strongly advocate for its routine adoption. Performance metrics should include common quantitative indicators such as AUC, accuracy, sensitivity, and specificity, complemented by point estimates with confidence intervals to reflect uncertainty, as well as calibration metrics like the Brier score. Interpretability techniques such as SHAP (SHapley Additive exPlanations) and LIME (Local Interpretable Model-agnostic Explanations) can be applied to elucidate the decision logic of the model and enhance transparency. Fairness audits across demographic subgroups (e.g. sex, ethnicity) should also be conducted to evaluate performance consistency. Beyond predictive performance, evaluating the clinical utility of models—including DCA and health economic evaluations, such as cost-utility analysis (CUA) and cost-effectiveness analysis (CEA, e.g. using the incremental cost-effectiveness ratio, ICER)—is also essential to quantify real-world benefits and provide early evidence of clinical value.

Prospective clinical validation represents the key step in bridging experimental success and clinical translation. Model performance and feasibility should be evaluated in real-world or prospective clinical trial settings, assessing adaptability and safety across diverse populations and healthcare environments. Establishing a ‘clinician-in-the-loop’ validation system can enhance interaction between algorithms and clinical experts, improving clinical credibility and acceptance.

To obtain clinical approval, AI models must undergo regulatory review. AI/ML models intended for use as software medical devices (SaMD) should comply with international regulatory frameworks, such as the U.S. Food and Drug Administration (FDA), the European Medicines Agency (EMA), and the National Medical Products Administration (NMPA), to ensure clinical performance, safety, and effective risk management. The regulatory process should encompass performance verification, ethical and fairness evaluation, data privacy and security protection, and submission of comprehensive documentation on risk and performance for compliance review and market approval.

Finally, regulatory clearance does not mark the end of the model lifecycle but rather the beginning of its long-term management. During the real-world recalibration stage, continuous monitoring of model outputs and corresponding clinical outcomes is essential. Detection of data drift, collection of user feedback, and periodic performance evaluation should guide model retraining or recalibration. Updated models should be re-approved under regulatory oversight to ensure long-term accuracy, safety, and effectiveness.

## Discussion

This review systematically summarizes the advances in AI-based predictive modeling for the three major diabetic complications, DR, DN, and CVD. It covers modeling strategies based on clinical variables, molecular omics features, and multimodal data integration, aiming to provide a comprehensive understanding of the current status, challenges, and future directions in this field. At the feature level, models constructed with clinical data such as EHR [148, 149], laboratory tests [150, 151], and imaging [152, 153] are the most widely used due to their high accessibility. These models are effective in capturing phenotypic characteristics and disease progression, showing robust performance in short-term risk stratification. However, their ability to detect early, subclinical, or mechanism-driven pathological changes remains limited. Comparative analyses across complications reveal differing key predictors: retinal imaging features are critical for DR [154], renal function indicators are highly specific to DN [155], while hypertension and dyslipidemia serve as strong predictors for CVD [156] but also overlap with DR and DN [157]. Omics data, including transcriptomics, proteomics, and metabolomics, have injected mechanistic insights into predictive modeling for diabetic complications [158–160]. These approaches are particularly valuable for identifying early biomarkers and disease subtypes, potentially enabling targeted intervention before irreversible damage occurs [161, 162].

Multimodal data integration strategies are increasingly being explored to enhance risk prediction for diabetic complications. For example, Shen et al. [104] showed that integrating metabolomic information with clinical variables substantially enhanced predictive performance of CVD, while Looker et al. [105] demonstrated that combining protein biomarkers with clinical covariates improved cardiovascular risk stratification in individuals with T2D. These observations underscore the potential of multimodal fusion in advancing precision prediction and management of diabetic complications. However, current research and practice in multimodal fusion still face critical technical hurdles. Clinical and omics data differ substantially in scale, distribution, and noise patterns. Designing effective cross-modal alignment mechanisms, unified representation learning frameworks, and normalization strategies remains essential for achieving deep integration. Moreover, multimodal models tend to exacerbate the ‘black-box’ nature of AI systems, further hindering clinical interpretability. Promising technological directions include explicit modeling of complex inter-modality relationships using graph neural networks, leveraging Transformer architectures and cross-modal attention mechanisms to capture long-range dependencies, and integrating causal inference frameworks to enhance interpretability and model robustness.

Algorithm selection should be closely aligned with data characteristics and task objectives. Traditional ML methods, such as SVM, RF, and XGBoost, perform well on structured clinical data and small to medium-sized datasets [163]. They offer strong interpretability and low computational demands, making them more feasible for clinical adoption. However, their ability to model ultra-high-dimensional data or complex temporal dependencies, such as sequential fundus images or longitudinal EHR data, is limited. DL shows substantial advantages in handling complex data modalities [164, 165], particularly in medical imaging and time-series analysis. In DR diagnosis, CNN-based models have achieved performance on par with or even surpassing expert ophthalmologists [166, 167], overcoming the constraints of manual feature engineering. Nevertheless, the ‘black-box’ nature [168], dependency on large labeled datasets, and high computational cost hinder their widespread clinical implementation. Currently, LLMs represent a rapidly emerging research paradigm in diabetic complication prediction, with growing studies exploring their potential lies in processing unstructured clinical text [169], enabling personalized health management via conversational agents [170], and supporting biomedical knowledge discovery [171]. In conclusion, the performance of AI models depends on multiple factors including task specificity, data modality, and sample size. Algorithm selection should be tailored to the application scenario to balance accuracy, interpretability, and practicality.

This review also highlights the substantial heterogeneity that limits coherent quantitative synthesis across studies. Current AI models for diabetic complications are developed under diverse study designs, data sources, and evaluation criteria, resulting in inconsistent performance metrics. Although AUC, accuracy, or F1-score are commonly reported, these metrics are often derived from distinct prediction horizons, population characteristics, and endpoint definitions, and some studies report ranges while others omit certain metrics, reflecting incomplete and heterogeneous reporting of evaluation results. For instance, prediction timeframes vary widely from 1 to 5 years, with some studies incorporating observation and buffer periods, while others rely on cross-sectional data only. In addition, the differences in cohort size, ethnicity composition, validation strategy (internal versus external), and center number further hinder direct cross-study comparison. These sources of heterogeneity underscore that readers should interpret reported results cautiously, as performance metrics may not be directly comparable across studies. It is also important to note that most of the included studies did not perform decision-analytic evaluations, which represents a common limitation across the literature. Moreover, the limited availability of source data and model codes further undermines the reproducibility of current AI models. Future research should strengthen data and model sharing in accordance with principles of transparency and reproducibility. It is also essential to establish standardized reporting and evaluation frameworks that clearly define prediction horizon, sample representativeness, validation type, outcome definition, and confidence intervals for performance metrics, while incorporating decision-analytic metrics wherever feasible. Such practices would improve transparency, comparability, and the interpretability of AI model evidence in diabetic complication prediction.

Translating high-performing AI models into clinically actionable tools remains a major challenge. To facilitate this process, we propose an actionable framework and clinical translation pathway of AI in diabetic complications, which outlines six key steps: data preparation and management, data partitioning and statistical analysis, model development and validation, prospective clinical evaluation, regulatory approval, and real-world monitoring and recalibration. Through this systematic, stepwise design, the roadmap not only provides researchers with clear guidance for model development and validation but also offers funders and policymakers a reference for prioritizing research directions, optimizing resource allocation, and accelerating translational impact. Moreover, the framework emphasizes reproducibility, robust validation, clinical utility assessment, and regulatory compliance, aiming to provide a feasible pathway for moving AI innovations from laboratory research into clinical practice and ultimately enhancing patient management and decision-making. By integrating these elements into a structured roadmap, we hope to offer a practical foundation for future studies, supporting the responsible and effective translation of AI technologies into real-world clinical applications.

Key Points

- This review provides an overview of artificial intelligence applications in predicting three major diabetic complications: diabetic retinopathy, diabetic nephropathy, and diabetic cardiovascular disease.
- This work explores modeling strategies built on clinical variables, medical imaging, and molecular omics, emphasizing the strengths and limitations of each data type and highlighting cross-scale data fusion as a promising new paradigm.
- The development of predictive modeling is traced from traditional machine learning to deep learning, and to large language models and agent-based systems, with a focus on their unique capabilities/limitations and suitable application scenarios.
- This work proposes a six-stage actionable framework for developing and translating AI models in diabetic complications, providing practical guidance from data preparation to clinical validation and real-world implementation.

## Supplementary Material

bbag083_Supplementary_tables

## Data Availability

No new datasets were generated in this work.
